# Iron promotes both ferroptosis and necroptosis in the early stage of reperfusion in ischemic stroke

**DOI:** 10.1016/j.gendis.2024.101262

**Published:** 2024-03-08

**Authors:** Bin Du, Zijie Deng, Kang Chen, Zhangzhong Yang, Junfen Wei, Liuyao Zhou, Jie Meng, Ying Cheng, Xin Tian, Qing-Zhang Tuo, Peng Lei

**Affiliations:** a Department of Neurology and State Key Laboratory of Biotherapy, National Clinical Research Center for Geriatrics, West China Hospital, Sichuan University, Chengdu, Sichuan 610041, China; b Department of Neurology, First Affiliated Hospital of Chongqing Medical University, Chongqing Key Laboratory of Neurology, Chongqing 400016, China

**Keywords:** Deferoxamine, Ferroptosis, Iron, Ischemic stroke, Necroptosis

## Abstract

Programmed cell death contributes to neurological damage in ischemic stroke, especially during the reperfusion stage. Several cell death pathways have been tested preclinically and clinically, including ferroptosis, necroptosis, and apoptosis. However, the sequence and complex interplay between cell death pathways during ischemia/reperfusion remains under investigation. Here, we unbiasedly investigated cell death pathways during ischemia/reperfusion by utilizing RNA sequencing analysis and immunoblot assays and revealed that ferroptosis and necroptosis occurred early post-reperfusion, followed by apoptosis. Ferroptosis inhibitor Liproxstatin-1 effectively inhibited necroptosis during reperfusion, while the necroptosis inhibitor Necrostatin-1 suppressed protein expression consistent with ferroptosis activation. Protein–protein interaction analysis and iron chelation therapy by deferoxamine mesylate indicate that iron is capable of promoting both ferroptosis and necroptosis in middle cerebral artery occlusion/repression modeled mice. Treatment of cells with iron led to a disruption in redox balance with activated necroptosis and increased susceptibility to ferroptosis. Collectively, these data uncovered a complex interplay between ferroptosis and necroptosis during ischemic stroke and indicated that multiple programmed cell death pathways may be targeted co-currently.

## Introduction

Ischemic stroke (IS) is a significant risk factor for both death and disability worldwide, with substantial global health implications.^1^^,^^2^ This is particularly crucial in the context of COVID-19, which significantly increases the risk of IS onset by eightfold for critically ill patients.^3^, ^4^, ^5^ Despite considerable progress in understanding the pathological processes of IS-induced cerebral injury, clinical management options are still limited.

In recent years, neuroprotective therapies aimed at modulating acute neuroprotection and facilitating long-term neuroregeneration following an IS have been extensively investigated. Several neuroprotective drugs with reliable results in preclinical IS models have been translated into clinical trials, including nerinetide (ClinicalTrials.gov ID: NCT04462536), 3K3A-APC (ClinicalTrials.gov ID: NCT05484154), Neu2000 (ClinicalTrials.gov ID: NCT05041010), and dabigatran (ClinicalTrials.gov ID: NCT03961334).^6^, ^7^, ^8^ These drugs undergoing phase III clinical trials primarily target three key pathological processes of IS, inflammatory response, oxidative stress, and excitotoxicity, all of which are closely linked to programmed cell death pathways.

Programmed cell death, such as ferroptosis, necroptosis, apoptosis, and pyroptosis, plays an essential role in maintaining cellular homeostasis by eliminating damaged and senescent cells^9^ and has been implicated in mediating neurologic damage during IS.^10^^,^^11^ However, little was reported to define whether these cell death pathways were activated independently or synergically during IS. One study showed that neuronal necroptosis could be followed by apoptosis after the reduction of TAK1 under ischemic conditions,^12^ highlighting the possibility of a coordinated system to mediate disease-related pathology. This has been seen in other diseases. For example, apoptosis has been shown to transit into necroptosis upon the inhibition of caspase-8 and its adapter FAS-associated death domain protein.^13^^,^^14^ In a mouse model of metabolic dysfunction-associated fatty liver disease, the ferroptosis inhibitor Liproxstatin-1 (Lip-1) not only inhibited ferroptosis but also blocked PANoptosis, a novel form of cell death that includes apoptosis, necroptosis, and pyroptosis, resulting in protection against metabolic dysfunction-associated fatty liver disease.^15^ Therefore, understanding the interplays of cell death pathways may provide further clues to cure IS.

In the present study, we mapped the time-dependent activation of programmed cell death pathways in the brains of mice that underwent cerebral ischemia-reperfusion. Through bioinformatic analysis, we identified differentially expressed genes (DEGs) and pathways related to programmed cell death, and then employed pharmacological interventions in mice and cell culture to investigate the crosstalk. These results may provide further insights contributing to developing precise treatment strategies for IS.

## Materials and methods

### Reagents and assay kits

Lip-1 (S7699), Necrostatin-1 (Nec-1, S8037), Necrostatin-1s (Nec-1s, S8641), SM-164 (S7089), and Z-VAD-FMK (S7023) were purchased from Selleck Chemical (USA). Deferoxamine mesylate (HY–B0988) was obtained from MedChemExpress (USA). Recombinant human TNF-α (No. C008) was obtained from Novoprotein Scientific, Inc. (China). The malondialdehyde (MDA) assay kit (ab233471) was acquired from Abcam (USA), while the lipid peroxidation (LPO) assay kit (A106-1-2) and superoxide dismutase (SOD) assay kit (A001-3-2) were obtained from Jianchen (China). Unless otherwise specified, all other chemicals and reagents were purchased from Sangon Biotech (China).

## Animals

We obtained adult male C57BL/6 mice (25–30 g) from Hfk Bioscience (China). The mice were kept in a specific pathogen-free facility under controlled conditions of temperature and humidity, with a 12-h light/dark cycle, and had free access to food and water before the experiments. To ensure proper adaptation to the breeding environment, the mice were allowed to acclimate for at least one week. This research received ethical support from the Institutional Guidelines of the Animal Care and Use Committee of 10.13039/501100004912Sichuan University, China (No. K2018071). We followed a randomized, double-blind experimental design for all animal experiments.

### Cerebral ischemia-reperfusion model

All surgeries were performed by a skilled researcher under aseptic conditions. As described previously,^16^ adult male mice were anesthetized with a mixture of isoflurane and air. A 2-cm incision was made in the anterior neck region. Left unilateral middle cerebral artery occlusion/repression (MCAO/R) was achieved by inserting a silicon rubber-coated nylon monofilament (RWD Life Science, China) into the internal artery through the common carotid artery. The monofilament was advanced 9–10 mm past the carotid bifurcation until a slight resistance was detected. The adequacy of MCAO/R was confirmed by monitoring cortical blood flow using a PeriCam PSI system (Perimed, Järfälla, Sweden). Animals were excluded if the mean ipsilateral laser speckle signal exceeded 30% of the pre-ischemic ipsilateral hemisphere baseline. After 1 h of occlusion, the filament was extracted, followed by 2 h, 6 h, and 24 h of reperfusion. Sham-operated mice underwent the same surgery except for filament insertion. Throughout the procedures, mice were kept on a warming pad.

### Drug treatment

Mice were randomly selected for treatment using WPS Office (v2019, Kingsoft Office Software, China). Lip-1 (10 mg/kg), Nec-1 (20 mg/kg), or deferoxamine mesylate (100 mg/kg) were administered simultaneously via intranasal pipette and intraperitoneal injection once a day for 7 days before the induction of focal ischemia in C57BL/6 mice. Furthermore, an additional administration was performed before reperfusion. The vehicle group for the Lip-1 injection was administered a mixture containing 2% DMSO, 40% PEG 300, 2% Tween 80, and 56% ddH_2_O. The vehicle group for the Nec-1 injection was administered a mixture containing 5% DMSO, 45% PEG 300, and 50% ddH_2_O. The vehicle group for the 77deferoxamine mesylate injection was administered saline only.

### Neurological assessment

Following surgery, neurological assessments were conducted by a blinded investigator, and the results were subsequently confirmed by a second blinded investigator. At 0 h, 6 h, and 24 h after cerebral ischemia-reperfusion, each mouse's neurological deficit was evaluated using a previously described five-point scale^16^: 0, no observable deficit; 1, right forelimb flexion; 2, decreased resistance to left lateral push (and right forelimb flexion) without circling; 3, circling to the right with the same behavior as grade 2; 4, severe rotation progressing into barreling, loss of walking or righting reflex.

### Infarct volume analysis

The infarct volume was calculated as previously described.^16^ Briefly, mice were sacrificed at 6 h and 24 h post-reperfusion, and their brains were rapidly removed and placed at −80 °C for 5 min. The brains were then cut into four 2-mm-thickness slices. The slices were stained with 1%, 2%, 3%, 5%-triphenyltetrazolium chloride (TTC) (Sigma, St. Louis, MO, USA) at 37 °C for 10 min and then fixed in 4% paraformaldehyde overnight. Digital images were taken, and the infarct volumes were analyzed blindly using Image J (Ver 1.53a, NIH, USA). The infarct area (white, unstained), ipsilateral hemisphere area (white, unstained, plus red brick, stained), and contralateral hemisphere area (red brick, stained) were assessed and measured in each section by an operator who was blinded to the experiment. The volume was determined by summing the representative areas across all sections, multiplying by the slice thickness, and subsequently correcting for edema. The corrected infarct volume was calculated as follows: corrected infarct volume = contralateral hemisphere volume − (ipsilateral hemisphere volume − infarct volume).

### Cell culture and viability assays

The Neuro-2a mouse neuroblastoma (N2a) cell line (CCL-131, American Type Culture Collection) and L929 mouse fibroblast cell line (CL-0137, Procell Life Science&Technology) were cultured in DMEM (Gibco, Thermo Fisher Scientific) supplemented with 10% fetal bovine serum (Gibco, Thermo Fisher Scientific) and 1% penicillin-streptomycin in a humid incubator at 37 °C with 5% CO_2_. Cells (passaging begins from the eighth generation) were seeded onto 96-well plates and then treated with the compounds after plating. Then, cell viability was assessed 24 h after treatment using an MTT cytotoxicity assay kit (M2003, Sigma).

### Flow cytometry

N2a cells were treated with 10 μM and 100 μM ferrous (II) ammonium sulfate (FAS) for 24 h. Then, the cells were collected and washed with Dulbecco's phosphate-buffered saline (Gibco). The cell lines were incubated with FerroOrange (F374, Dojindo), the reactive oxygen species (ROS) assay kit (S0033, Beyotime), and BODIPY 581/591 C11 (D3861, Thermo Fisher Scientific). Subsequently, they were analyzed using different lasers of a flow cytometer (LSR Fortessa, BD) for excitation, following the manufacturer's guidelines.

### Immunoblotting

The procedures were performed as previously described.^17^ The samples were homogenized in cell lysis buffer (P0013, Beyotime, China), containing the protease inhibitor phenylmethylsulfonyl fluoride (ST507, Beyotime, China). The resulting mixture was then centrifuged at 12,000 *g* at 4 °C for 30 min. After centrifugation, the supernatant was collected, and the total protein concentration was measured using a BCA protein assay kit (P0011, Beyotime, China). Previous studies^12^^,^^18^ have demonstrated that necroptotic biomarkers exhibit differential solubilities upon activation. Therefore, to detect these biomarkers, we successively lysed proteins using a buffer containing Nonidet P-40 and 6M urea. Equal amounts of protein were loaded onto 4%–20% bis-Tris gels with MOPs running buffer and subjected to electrophoresis at 140 V for 1 h. The separated proteins were then transferred to polyvinylidene fluoride membranes using a Trans-Blot system at 100 V for 1 h. The membranes were washed with tris-buffered saline with Tween® 20 (TBST, 0.02 M, pH 8.0) at room temperature (20 °C) for 5 min, followed by blocking with 5% skim milk in TBST (0.02 M, pH 8.0) at room temperature for 1 h. The membranes were subsequently incubated with primary antibody diluted in TBST (0.02 M, pH 8.0) at 4 °C overnight while being rocked. The next day, the membranes were washed five times for 5 min each in TBST (0.02 M, pH 8.0) and then incubated with peroxidase-conjugated secondary antibody diluted in TBST (0.02 M, pH 8.0) at room temperature for 120 min while being rocked. After incubation with the secondary antibody, the membranes were washed three times for 20 min each in TBST (0.02 M, pH 8.0) and then subjected to enhanced chemiluminescence (P10300, NCM Biotech, China) development. Finally, the membranes were visualized using a ChemiScope 6100 (CLiNX, China), and the chemiluminescence band intensity was quantified using Image J (Ver 1.53a, NIH, USA).

In this study, the following antibodies were diluted in TBST (0.02 M, pH 8.0) as indicated: 1:5000 for anti-ACSL4 (ab155282, Abcam, USA); 1:5000 for anti-GPX4 (ab125066, Abcam, USA); 1:5000 for anti-SLC7a11 (ab175186, Abcam, USA); 1:1000 for anti-RIPK1 (3493S, Cell Signaling Technology, USA); 1:1000 for anti-Phospho-RIPK1 (Ser166) (31122S, Cell Signaling Technology, USA); 1:1000 for anti-RIPK3 (95702S, Cell Signaling Technology, USA); 1:1000 for anti-Phospho-RIPK3 (Thr231/Ser232) (91702S, Cell Signaling Technology, USA); 1:1000 for anti-MLKL (37705S, Cell Signaling Technology, USA); 1:1000 for anti-Phospho-MLKL (Ser345) (Cell Signaling Technology, USA, 37333S); 1:1000 for anti-Cleaved Caspase-3 (9664S, Cell Signaling Technology, USA); 1:10000 for anti-mouse Ig (A9044, Sigma–Aldrich, USA); 1:10,000 for anti-rabbit Ig (A0545, Sigma Aldrich, USA); 1:10000 for anti-β-actin (A5441, Sigma–Aldrich, USA); 1:10000 for anti-GAPDH (HRP-60004, Proteintech, USA).

### RNA-seq analysis

RNA samples were collected from the cortex of IS subjects with different reperfusion times as indicated in the figures. Total RNA was isolated from the tissues using TRIzol reagent (15596026, Thermo Fisher). Quantity and purity were analyzed with Bioanalyzer 2100 and RNA 6000 Nano LabChip Kit (Agilent, CA, USA) with a RIN score >7.0 as the cutoff. Approximately 1 μg of total RNA representing a specific adipose type was subjected to isolate poly(A) mRNA with poly-T oligo-attached magnetic beads. Following purification, the mRNA was fragmented into ∼600 nt long oligonucleotides using divalent cations under elevated temperature. The cleaved RNA fragments were reverse-transcribed to create the final cDNA library by the protocol for the mRNA Seq sample preparation kit (Illumina, San Diego, USA) by the dUTP method, and the average insert size for the paired-end libraries was 300 bp (±50 bp). Subsequent paired-end 2 × 150 bp (PE150) sequencing was performed on an Illumina Novaseq 6000 platform at Mega Genomics (Beijing, China) following the vendor's recommended protocol. There were five replicates per experimental condition.

DEGs were determined using the limma software package analysis,^19^ with fold change >1.5 and *P* < 0.05 as DEGs with statistically significant differences. Volcano plots and heatmaps were generated using “ggplot2” and the “pheatmap” packages, showing statistically significant DEGs. To further reveal the important functions and biological pathways of DEGs, we performed the Kyoto Encyclopedia of Genes and Genomes (KEGG) pathway enrichment analysis on them. The “clusterProfiler” and “ggplot2” packages were used to analyze and visualize the KEGG pathway enrichment of differential genes. *P* < 0.05 and false discovery rate (FDR) < 0.25 were considered statistically significant. Gene Set Enrichment Analysis (GSEA) (version 4.0.3, www.broadinstitute.org/gsea/) was used to identify the enrichment of cell death pathways. *P* < 0.05, FDR <0.25, and |normalized enrichment score| >1 were considered statistically significant. Protein–protein interaction network was constructed using the Search Tool for the Retrieval of Interacting Genes/Proteins (STRING, https://string-db.org/).^20^ Subsequently, the results of STRING were analyzed and visualized using Cytoscape software (version 3.9.0).^21^ All bioinformatic analyses were conducted using the R environment (version 3.5.2, R Foundation, Austria).

### Statistical analysis

Individual values are displayed in the data. The statistical analysis was conducted using GraphPad Prism software (Ver 8.0 for Windows, GraphPad Software, USA) and the results were presented as mean ± standard error of the mean. The figure legends contain descriptions of the statistical methods used. Significance was determined by *P*-values less than 0.05.

## Results

### Activation of ferroptosis, necroptosis, and apoptosis in the brain post-reperfusion

To investigate the time sequence of cell death activation during IS, we first established the cerebral ischemia-reperfusion model, which involved 60 min of occlusion followed by 6 h and 24 h of reperfusion. TTC staining revealed significant increases in infarct volume at 6 h and 24 h post-MCAO/R (Fig. S1A, B), along with neurological scores (Fig. S1C).

**Figure 1:**
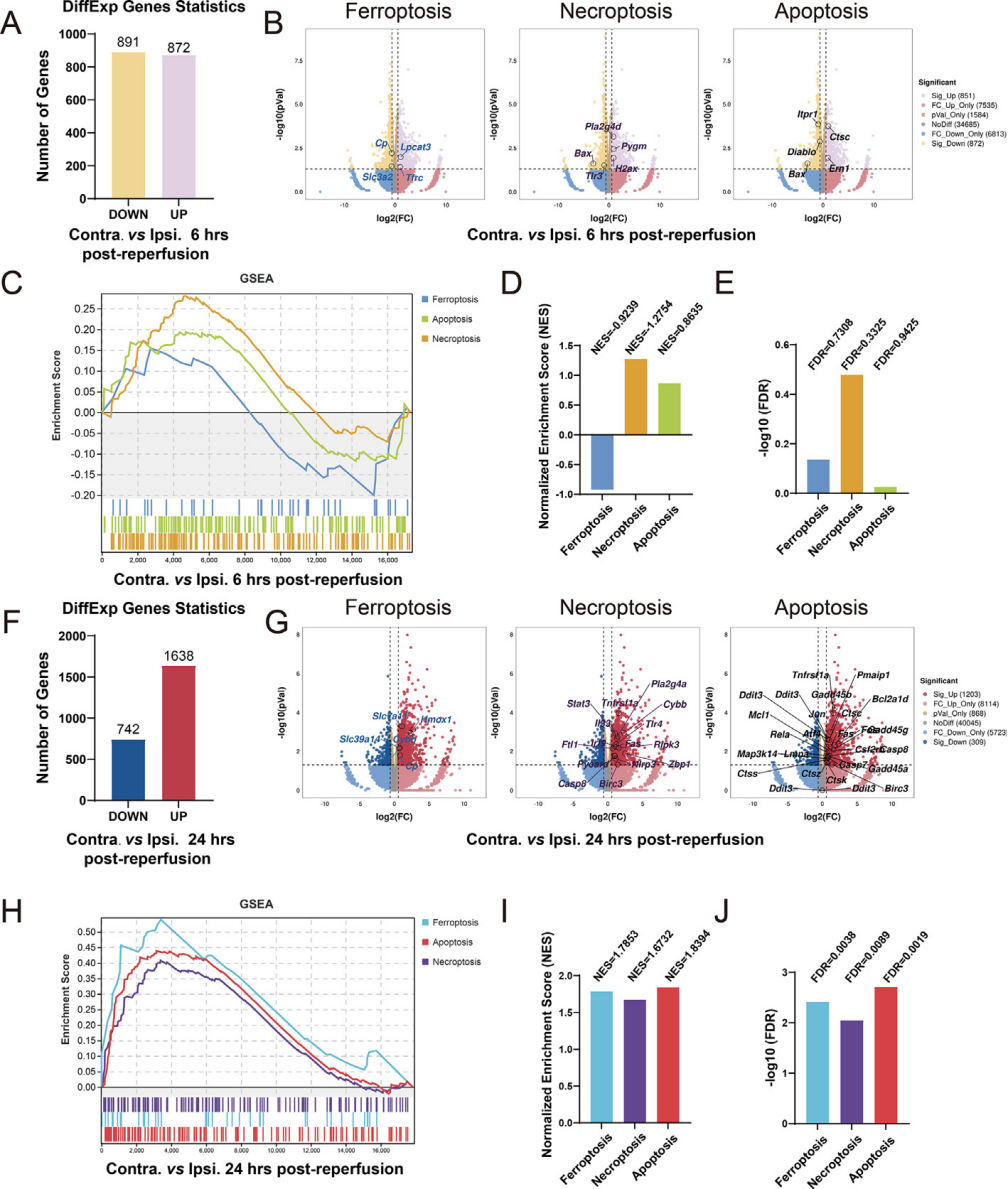
Ischemic stroke leads to significant changes in cell death-related genes and pathways within the cortex during reperfusion. **(A)** The number of DEGs between the ipsilesional and contralesional sides at 6 h post-reperfusion. **(B)** Volcano plot of DEGs associated with cell death between the ipsilesional and contralesional sides at 6 h post-reperfusion. **(C)** GSEA enrichment analysis associated with cell death pathways for comparing the ipsilesional side with the contralesional side at 6 h post-reperfusion. **(D, E)** The GSEA analysis revealed representative NES (D) and FDR values (E), highlighting their association with cell death pathways at 6 h post-reperfusion. The data underwent analysis using R software, with *n* = 5 animals per group. **(F)** The number of DEGs between the ipsilesional and contralesional sides at 24 h post-reperfusion. **(G)** Volcano plot of DEGs associated with cell death between the ipsilesional and contralesional sides at 24 h post-reperfusion. **(H)** GSEA enrichment analysis associated with cell death pathways for comparing the ipsilesional side with the contralesional side at 24 h post-reperfusion. **(I, J)** The GSEA analysis revealed representative NES (I) and FDR values (J), highlighting their association with cell death pathways at 24 h post-reperfusion. The data underwent analysis using R software, with *n* = 4 animals per group. DEGs, differentially expressed genes; FDR, false discovery rate; GSEA, gene set enrichment analysis; NES, normalized enrichment score.

We then performed unbiased RNA-seq analysis using the cortical tissue of mice in this experiment. A total of 1763 DEGs were identified when the tissues were collected 6 h post-reperfusion, with 872 up-regulated and 891 down-regulated (Fig. 1A). The KEGG analysis, encompassing all pathways, revealed 27 significantly enriched pathways among the DEGs, with the top 5 pathways being hypertrophic cardiomyopathy, oxytocin signaling pathway, adrenergic signaling in cardiomyocytes, proteoglycans in cancer, and aldosterone synthesis and secretion (Fig. S1D). Several of these pathways implicate the occurrence of cardiovascular events as proposed previously.^22^ On the other hand, the KEGG pathway enrichment analysis, which focused on cellular processes, revealed seven pathways associated with cell growth and death. These pathways include the p53 signaling pathway, cellular senescence, cell cycle, oocyte meiosis, ferroptosis, necroptosis, and apoptosis (Fig. S1E). Therefore, we specifically concentrated on ferroptosis, necroptosis, and apoptosis in follow-up analysis.

We then analyzed these DEGs for their overlap with cell death-related genes obtained from the Reactome Pathway Database (https://reactome.org/).^23^ This analysis revealed 4 ferroptosis-related genes, 5 necroptosis-related genes, and 5 apoptosis-related genes (Fig. 1B). However, the GSEA enrichment analysis showed that these three cell death pathways were not significantly enriched (|normalized enrichment score| < 1 or FDR >0.25) (Fig. 1C–E), similar to the KEGG pathway analysis (Fig. S1E). These findings demonstrate that while there were changes in the genes related to cell death pathways at 6 h post-reperfusion, the pathways themselves still lack significant enrichment.

In contrast, 2380 DEGs were identified 24 h post-reperfusion. Among these, 1638 were up-regulated and 742 were down-regulated (Fig. 1F), including 6 ferroptosis-related genes, 15 necroptosis-related genes, and 23 apoptosis-related genes (Fig. 1G). The KEGG enrichment analysis revealed 84 significantly enriched pathways among the 2380 DEGs, with the TNF signaling pathway, osteoclast differentiation, complement and coagulation cascades, ECM-receptor interaction, and cytokine–cytokine receptor interaction pathways being the top 5 (Fig. S1F). Interestingly, the majority of these pathways are intimately associated with inflammation, establishing a strong correlation with cell death.^24^^,^^25^ The KEGG analysis focusing on cellular processes revealed a heightened level of enrichment in ferroptosis, necroptosis, and apoptosis (Fig. S1G). Similarly, GSEA analysis indicated that all three cell death pathways were significantly enriched (|normalized enrichment score| > 1 and FDR <0.25) (Fig. 1H–J). Taken together, our findings indicate that the response of cell death pathways undergoes significant changes over time in cerebral ischemia-reperfusion injury.

### Activations of both ferroptosis and necroptosis are early events during cerebral ischemia-reperfusion

**Figure 2:**
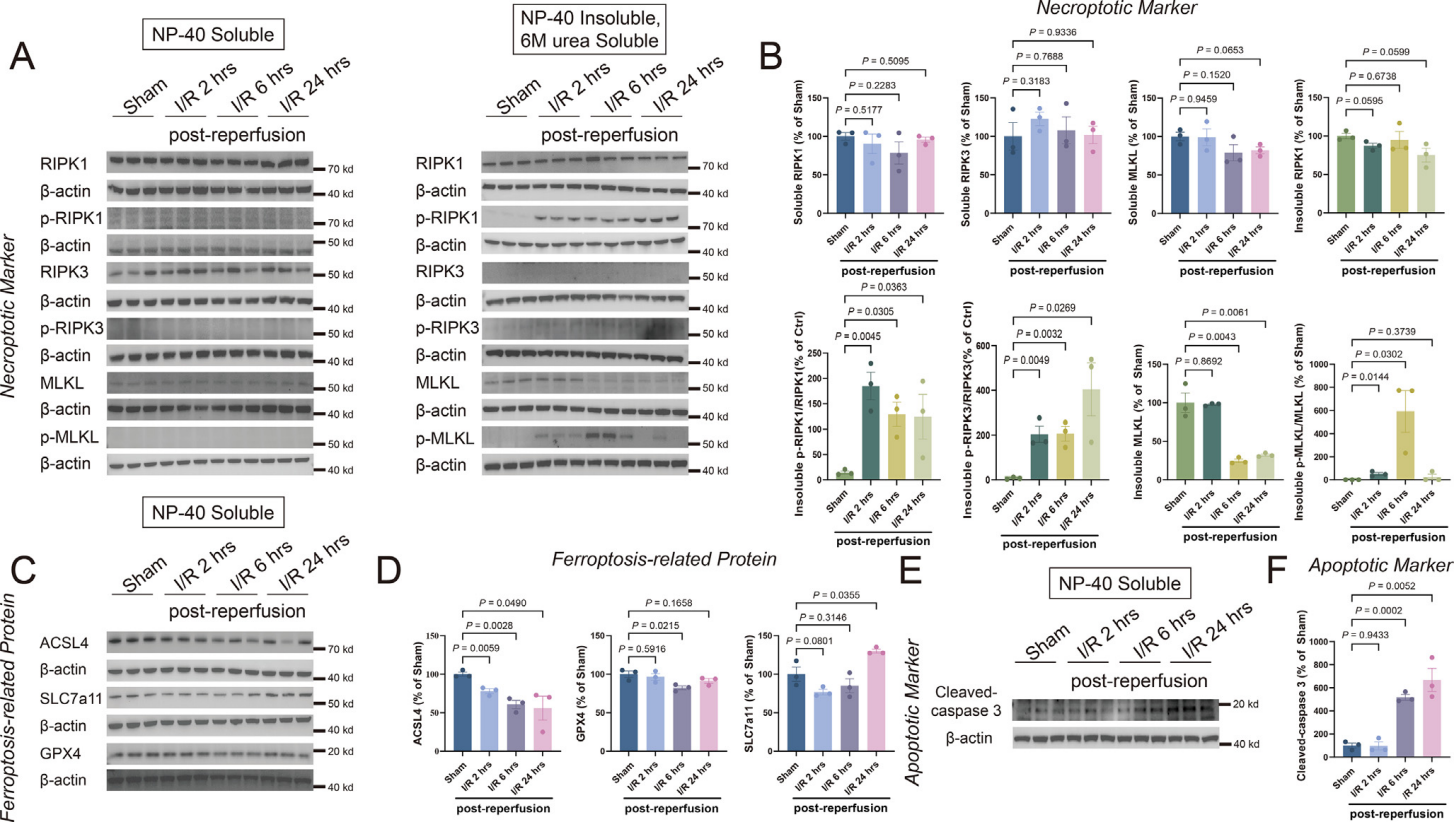
Sequential activation of ferroptosis, necroptosis, and apoptosis during reperfusion. **(A, B)** The levels of necroptotic markers, including soluble and insoluble components, were analyzed from the ischemic ipsilateral cortex of mice that underwent reperfusion at 2 h, 6 h, or 24 h. Western blots were assessed using Image J and normalized to β-actin expression. The statistical graph depicted the data concerning the necroptotic markers. **(C, D)** The levels of ferroptosis-related proteins were analyzed from the ischemic ipsilateral cortex of mice that underwent reperfusion at 2 h, 6 h, or 24 h. Western blots were assessed using Image J and normalized to β-actin expression. The statistical graph depicted the data concerning the ferroptosis-related proteins. **(E, F)** The levels of apoptotic markers were analyzed from the ischemic ipsilateral cortex of mice that underwent reperfusion at 2 h, 6 h, or 24 h. Western blots were assessed using Image J and normalized to β-actin expression. The statistical graph depicted the data concerning the apoptotic marker. The data presented are mean ± standard error of the mean, with *n* = 3 animals per group. One-way ANOVA with a post-hoc Tukey test was performed.

One limitation of RNA-seq analysis is that certain vital phenomena may have been overlooked or excluded.^26^ To validate the RNA sequencing analyses, we have systematically examined the protein expression of cell death pathways in cerebral ischemia-reperfusion injury. Here, we introduced a new group that collected 2 h post-reperfusion to study the time sequence. We firstly assessed the expression of biomarkers for necroptosis, including RIPK1, RIPK3, and MLKL, as well as their active forms p-RIPK1, p-RIPK3, and p-MLKL (Fig. 2A). During necroptosis, the activation of RIPK1 leads to increased phosphorylation of p-RIPK3, which further phosphorylates MLKL.^27^^,^^28^ While RIPK1 was unaltered in both soluble and insoluble fractions, p-RIPK1, a biomarker of RIPK1 activation, increased rapidly in the insoluble fraction 2 h post-reperfusion (Fig. 2B). In line with this, there were immediate detections of insoluble p-RIPK3 and p-MLKL 2 h post-reperfusion, where this elevation was persisted until 6 h post-reperfusion (Fig. 2B). These findings collectively indicate that necroptosis was activated in the early-stage post-reperfusion.

Despite the lack of established markers, we examined proteins relevant to ferroptosis, including ACSL4, SLC7a11, and GPX4, in the cortex following MCAO/R (Fig. 2C). Lowered levels of GPX4 were observed 6 h post-reperfusion, which then elevated to normal levels 24 h post-reperfusion. SLC7a11 was significantly reduced 2 h post-reperfusion and up-regulated 24 h post-reperfusion. On the other hand, a continuous reduction in ACSL4 to 24 h post-reperfusion was observed (Fig. 2D), consistent with our previous studies.^29^

The key biomarker for apoptosis, the cleavage product of caspase-3, was barely detectable at 2 h post-reperfusion. However, it was detectable at 6 h post-reperfusion and reached its peak at 24 h post-reperfusion (Fig. 2E, F). Therefore, in contrast to the changes observed in ferroptosis and necroptosis at 2 h post-reperfusion, apoptosis occurred with a delay.

We hypothesized that cell death pathways were mainly activated during the reperfusion stage, and established a permanent occlusion stroke model to test. No significant changes were found in ACSL4, SLC7A11, and GPX4, nor insoluble p-MLKL (Fig. S2A, B), indicating that reperfusion following ischemia is critical for the initiation of cell death pathways as hypothesized. These findings demonstrate an occurrence of ferroptosis and necroptosis during reperfusion injury, followed by apoptosis.

### Interactions between ferroptosis and necroptosis during reperfusion

Our data here indicate that ferroptosis and necroptosis were activated early during cerebral ischemia-reperfusion injury. Considering a previous report showing that neuronal necroptosis can transit into apoptosis,^17^ we investigated the potential interactions between ferroptosis and necroptosis.

**Figure 3:**
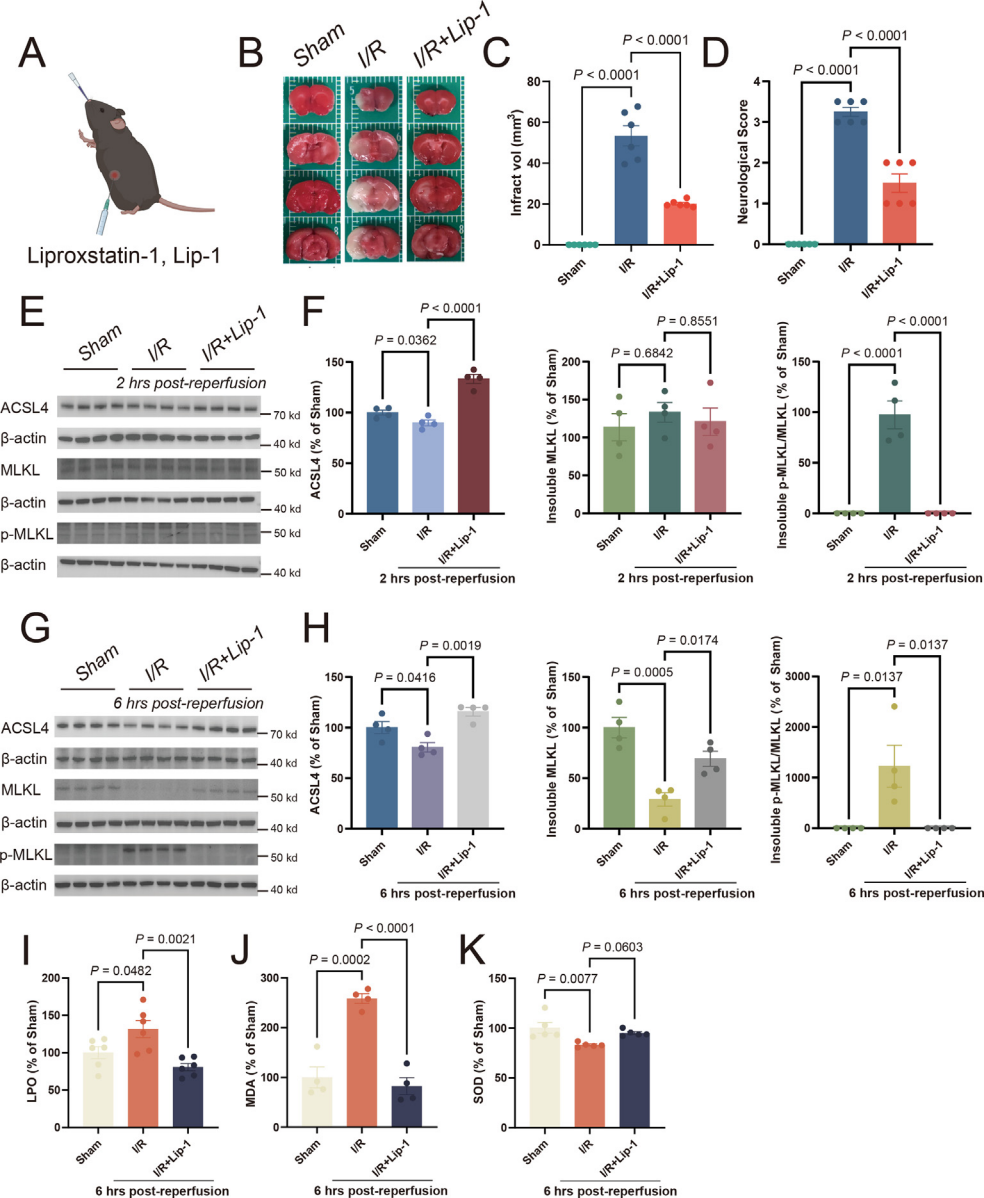
The ferroptosis inhibitor Liproxstatin-1 inhibits necroptosis during reperfusion. **(A)** Illustration of Liproxstatin-1 administration. **(B, C)** After Liproxstatin-1 administration, representative consecutive brain sections of MCAO/R mice were stained with TTC at 24 h post-reperfusion, where viable tissue was stained red. The infarction volume indicated by TTC staining was quantified using Image J. The data presented are mean ± standard error of the mean (SEM). *n* = 6 animals per group. One-way ANOVA with a post-hoc Tukey test was performed. **(D)** After Liproxstatin-1 administration, neurological scoring was performed at 24 h post-reperfusion. A higher number of scores indicates more damage. The data presented are mean ± SEM, with *n* = 6 animals per group. One-way ANOVA with a post-hoc Tukey test was performed. **(E, F)** After Liproxstatin-1 administration, the levels of ACSL4, insoluble MLKL, and insoluble p-MLKL were analyzed from the ischemic ipsilateral cortex of mice that underwent ischemia at 2 h post-reperfusion. Western blots were assessed using Image J and normalized to β-actin expression. The statistical graph depicted the data concerning the changes in protein expression at 2 h post-reperfusion. The data presented are mean ± SEM, with *n* = 4 animals per group. One-way ANOVA with a post-hoc Tukey test was performed. **(G, H)** After Liproxstatin-1 administration, the levels of ACSL4, insoluble MLKL, and insoluble p-MLKL were analyzed from the ischemic ipsilateral cortex of mice that underwent ischemia at 6 h post-reperfusion. Western blots were assessed using Image J and normalized to β-actin expression. The statistical graph depicted the data concerning the changes in protein expression at 6 h post-reperfusion. The data presented are mean ± SEM, with *n* = 4 animals per group. One-way ANOVA with a post-hoc Tukey test was performed. **(I–K)** After Liproxstatin-1 administration, the levels of LPO (I), MDA (J), and SOD (K) in the cortex were assayed following MCAO/R for 6 h. The data presented are mean ± SEM, with *n* = 6 animals per group (LPO), *n* = 4 animals per group (MDA), and *n* = 5 animals per group (SOD). One-way ANOVA with a post-hoc Tukey test was performed. MCAO/R, middle cerebral artery occlusion/repression; TTC, 2, 3, 5-triphenyltetrazolium chloride.

Lip-1, a ferroptosis inhibitor, can reduce infarct volumes and improve neurological functions within 24 h of MCAO/R (Fig. 3A–D), consistent with our earlier report.^16^ Lip-1 injection induced up-regulation of ACSL4 as early as 2 h post-reperfusion (Fig. 3E), indicating the importance of ACSL4. Unexpectedly, Lip-1 also inhibited the elevation of insoluble p-MLKL (a biomarker of necroptosis) at 2 h and 6 h post-reperfusion (Fig. 3E–H). Antioxidant status was assessed by measuring LPO, MDA, and SOD in tissue. Given the occurrence of ferroptosis and necroptosis during the early phase of reperfusion, with notable manifestation at 6 h post-reperfusion, our primary focus was on evaluating the changes specifically at this time point. We found that, following MCAO/R in mice (6 h post-reperfusion), LPO and MDA levels significantly increased and SOD levels significantly decreased in the affected cortical regions of the ipsilesional side compared with the contralesional side. The increase of LPO and MDA levels can be inhibited by Lip-1 treatment (Fig. 3I, J), as reported previously,^29^ while SOD level was not affected (Fig. 3K). Therefore, inhibition of ferroptosis by Lip-1 prevented the disruption of redox balance and necroptosis, both of which may contribute to the rescue effect of the compound.

**Figure 4:**
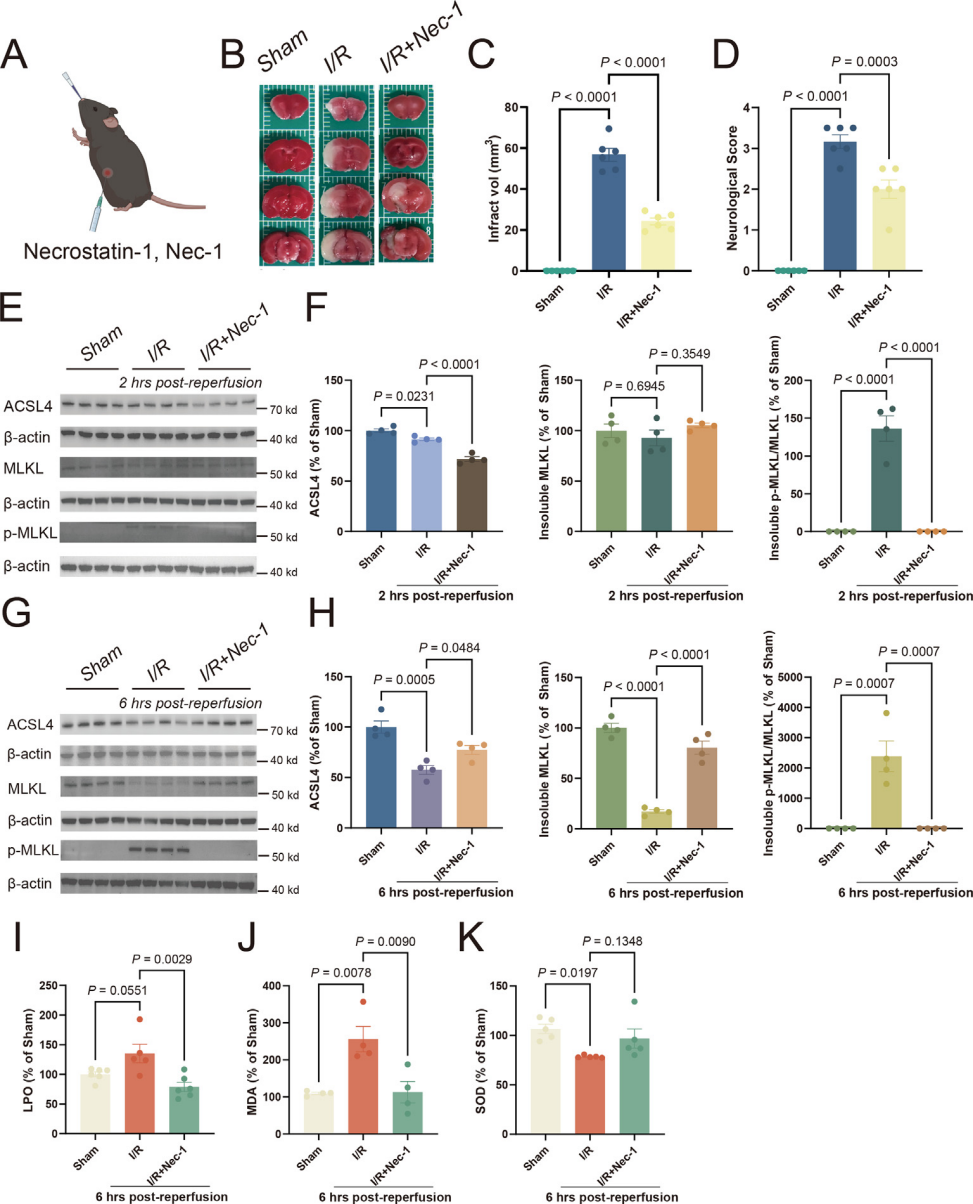
The necroptosis inhibitor Necrostatin-1 affects ferroptosis during reperfusion. **(A)** Illustration of Necrostatin-1 administration. **(B, C)** After Necrostatin-1 administration, representative consecutive brain sections of MCAO/R mice stained with TTC at 24 h post-reperfusion, where viable tissue stains red (B). Quantification of infarction volume indicated by TTC staining using Image J (C). Data are means ± standard error of the mean (SEM). *n* = 6 animals per group. One-way ANOVA with a post-hoc Tukey test was performed. **(D)** After Necrostatin-1 administration, neurological scoring was performed 24 h post-reperfusion. Data are means ± SEM, *n* = 6 animals per group. One-way ANOVA with a post-hoc Tukey test was performed. **(E, F)** After Necrostatin-1 administration, the levels of ACSL4, insoluble MLKL, and insoluble p-MLKL were analyzed from the ischemic ipsilateral cortex of mice that underwent ischemia at 2 h post-reperfusion (E). Western blots were assessed using Image J and normalized to β-actin expression. The statistical graph depicted the data concerning the changes in protein expression at 2 h post-reperfusion (F). The data presented are means ± SEM, with *n* = 4 animals per group. One-way ANOVA with a post-hoc Tukey test was performed. **(G, H)** After Necrostatin-1 administration, the levels of ACSL4, insoluble MLKL, and insoluble p-MLKL were analyzed from the ischemic ipsilateral cortex of mice that underwent ischemia at 6 h post-reperfusion (G). Western blots were assessed using Image J and normalized to β-actin expression. The statistical graph depicted the data concerning the changes in protein expression at 6 h post-reperfusion (H). The data presented are means ± SEM, with *n* = 4 animals per group. One-way ANOVA with a post-hoc Tukey test was performed. **(I–K)** After Necrostatin-1 administration, the levels of LPO (I), MDA (J), and SOD (K) in the cortex were assayed following MCAO/R for 6 h. Data are means ± SEM, *n* = 6 animals per group (LPO), *n =* 4 animals per group (MDA), and *n* = 5 animals per group (SOD). One-way ANOVA with a post-hoc Tukey test was performed. LPO, lipid peroxidation; MCAO/R, middle cerebral artery occlusion/repression; MDA, malondialdehyde; SOD, superoxide dismutase.

We also tested the necroptosis inhibitor Nec-1, which similarly resulted in the reduction of infarct volumes and improvement of neurological function 24 h post-reperfusion (Fig. 4A–D). Nec-1 inhibited the expression of the necroptosis biomarker p-MLKL at 2 h and 6 h post-reperfusion, and the treatment surprisingly promoted the reduction of ACSL4 at 2 h post-reperfusion, while preventing the further decrease at 6 h post-reperfusion (Fig. 4E–H). It also restored the affected LPO and MDA levels (Fig. 4I, J), while SOD was unaffected (Fig. 4K). Therefore, it is likely that inhibition of necroptosis affects the execution of ferroptosis.

### Iron impacts both ferroptosis and necroptosis during reperfusion

**Figure 5:**
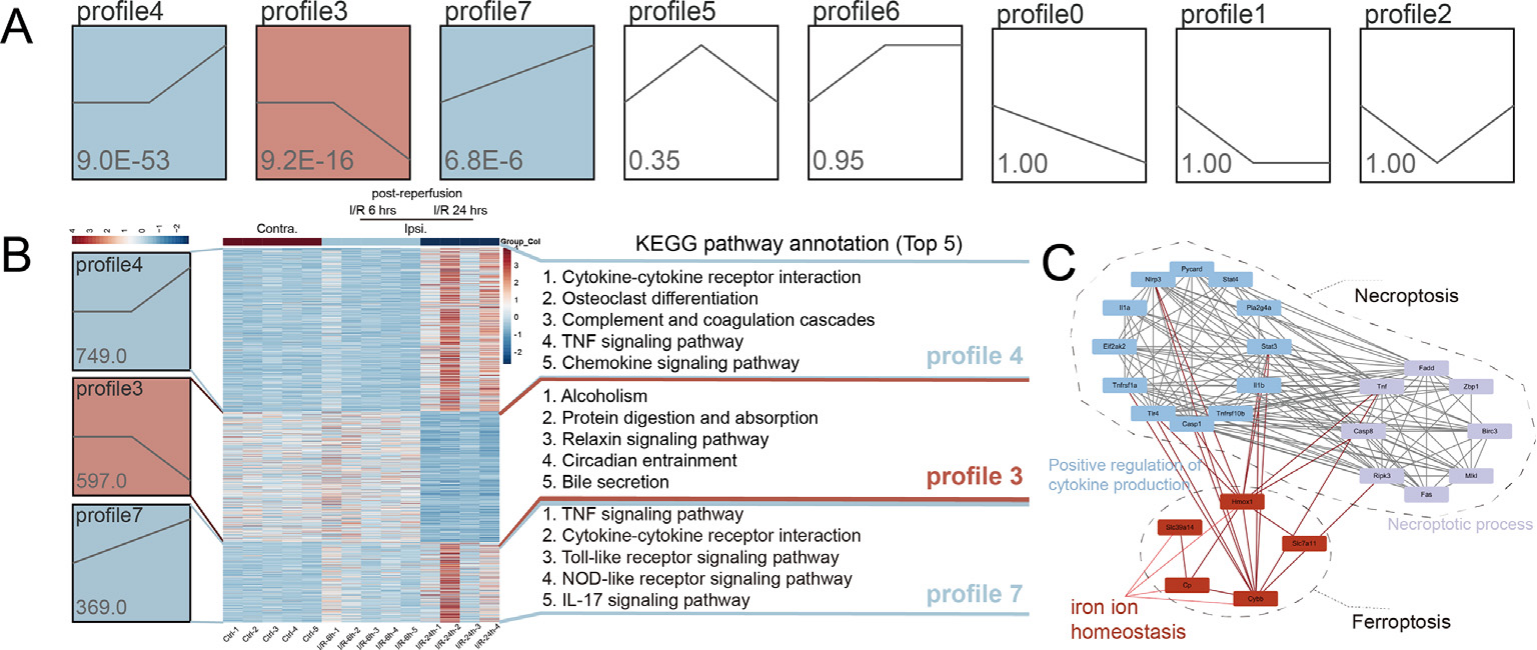
Iron may interact with both ferroptosis and necroptosis. **(A)** The statistically significant profiles by trend analysis of genes at contralesional and ipsilesional 6 h and 24 h post-reperfusion. The profiles that are colored represent statistically significant differences (*P* < 0.05). **(B)** Heatmap of genes in the three profiles. Columns represent samples and rows represent genes. For each profile gene, the text shows the top 5 pathways identified through KEGG enrichment analysis. **(C)** The crosstalk points between ferroptosis and necroptosis were identified through protein–protein interaction network analysis.

To investigate the potential mechanisms that inhibition of ferroptosis spontaneously inhibits necroptosis during IS, we performed a trend analysis on RNA-seq results over time post-reperfusion and identified eight gene expression profiles. Three of those profiles exhibited significance (*P* < 0.05): profile 4 (an upward trend beginning 6 h post-reperfusion), profile 3 (a downward trend beginning 6 h post-reperfusion), and profile 7 (continuously increased from control) (Fig. 5A). We then identified the top five KEGG pathways for each profile (Fig. 5B). In profile 4 which included 749 genes, the enriched pathways include cytokine–cytokine receptor interaction, osteoclast differentiation, complement and coagulation cascades, TNF signaling pathway, and chemokine signaling pathway. In profile 3 which included 597 genes, alcoholism, protein digestion and absorption, relaxin signaling pathway, circadian entrainment, and bile secretion were identified. In profile 7 which included 369 genes, the pathways of TNF signaling, cytokine–cytokine receptor interaction, Toll-like receptor signaling, NOD-like receptor signaling, and IL-17 signaling were enriched (Fig. 5B). It is noteworthy that pathways such as the TNF signaling pathway, NOD-like receptor signaling pathway, and complement and coagulation cascades have been reported to play a role in ferroptotic and necroptotic processes.^25^^,^^27^^,^^30^, ^31^, ^32^

We, therefore, utilized significant three profiles from trend analysis to identify genes linked with ferroptosis and necroptosis, to calculate the protein–protein interaction network. We identified the iron-related pathways as a crucial link between ferroptosis and necroptosis (Fig. 5C), indicating that iron likely plays a significant role in the interplay between these pathways.

**Figure 6:**
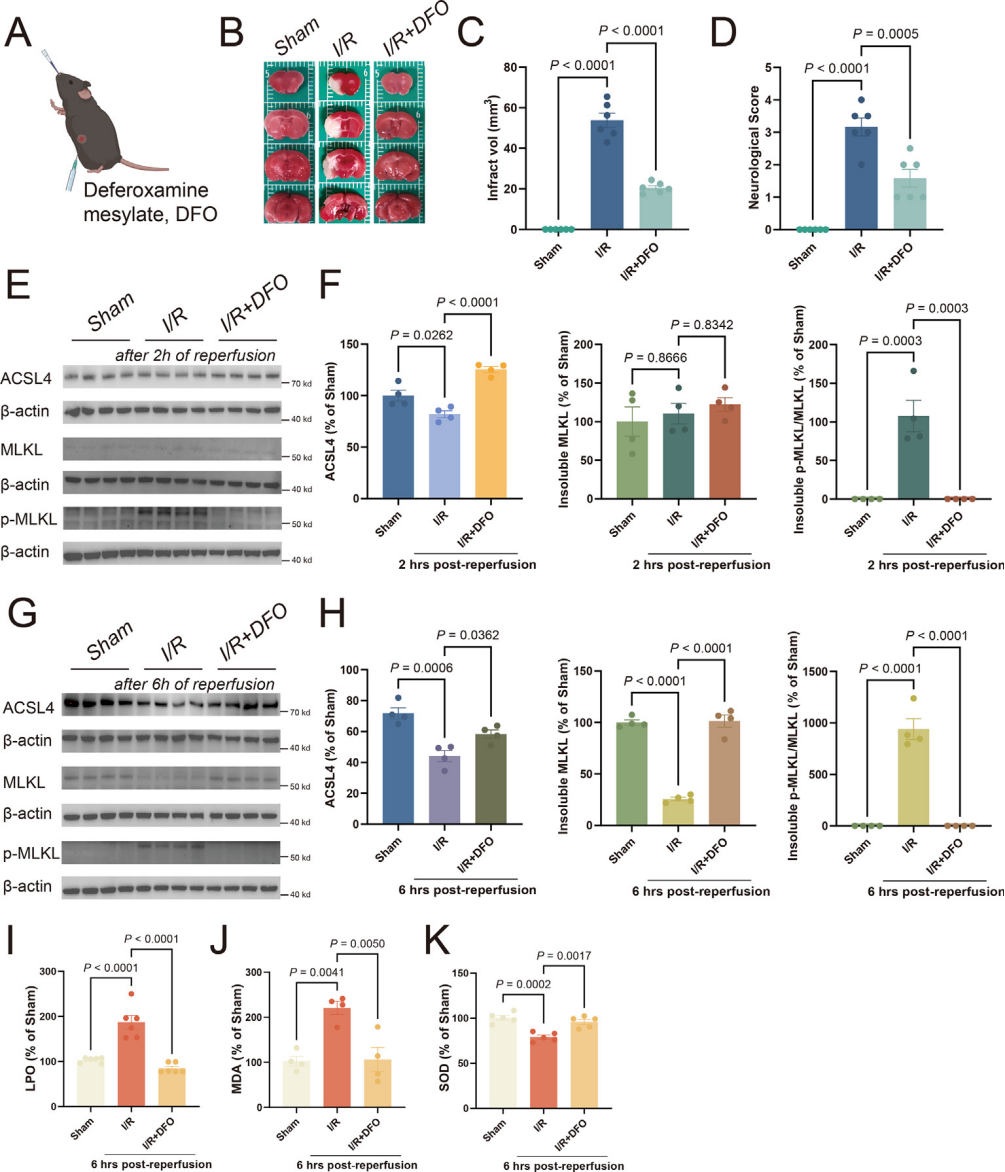
The iron chelator deferoxamine mesylate suppressed both ferroptosis and necroptosis during reperfusion. **(A)** Illustration of deferoxamine mesylate (DFO) administration. **(B, C)** After DFO administration, representative consecutive brain sections of MCAO/R mice were stained with TTC at 24 h post-reperfusion, where viable tissue was stained red. The infarction volume indicated by TTC staining was quantified using Image J. The data presented are mean ± standard error of the mean (SEM), with *n* = 6 animals per group. One-way ANOVA with a post-hoc Tukey test was performed. **(D)** After DFO administration, neurological scoring was performed 24 h post-reperfusion. The data presented are mean ± SEM, with *n* = 6 animals per group. One-way ANOVA with a post-hoc Tukey test was performed. **(E, F)** After DFO administration, the levels of ACSL4, insoluble MLKL, and insoluble p-MLKL were analyzed from the ischemic ipsilateral cortex of mice that underwent ischemia at 2 h post-reperfusion. Western blots were assessed using Image J and normalized to β-actin expression. The statistical graph depicted the data concerning the changes in protein expression at 2 h post-reperfusion. The data presented are mean ± SEM, with *n* = 4 animals per group. One-way ANOVA with a post-hoc Tukey test was performed. **(G, H)** After DFO administration, the levels of ACSL4, insoluble MLKL, and insoluble p-MLKL were analyzed from the ischemic ipsilateral cortex of mice that underwent ischemia at 6 h post-reperfusion. Western blots were assessed using Image J and normalized to β-actin expression. The statistical graph depicted the data concerning the changes in protein expression at 6 h post-reperfusion. The data presented are mean ± SEM, with *n* = 4 animals per group. One-way ANOVA with a post-hoc Tukey test was performed. **(I–K)** After DFO administration, the levels of LPO (I), MDA (J), and SOD (K) in the cortex were assayed following MCAO/R for 6 h. The data presented are mean ± SEM, with *n* = 6 animals per group (LPO), *n* = 4 animals per group (MDA), and *n* = 5 animals per group (SOD). One-way ANOVA with a post-hoc Tukey test was performed. LPO, lipid peroxidation; MCAO/R, middle cerebral artery occlusion/repression; MDA, malondialdehyde; SOD, superoxide dismutase; TTC, 2, 3, 5-triphenyltetrazolium chloride.

To verify, we evaluated whether iron chelator deferoxamine mesylate (DFO) could inhibit both ferroptosis and necroptosis during IS (Fig. 6A). As previously reported,^33^ within 24 h of MCAO/R, DFO prevented the increased infarct volume and improved neurological functions (Fig. 6B–D). DFO treatment also rescued the reduction in ACSL4 at 2 h and 6 h post-reperfusion (Fig. 6E–H). Interestingly, DFO treatment strongly inhibited necroptosis, evidenced by the significantly reduced insoluble p-MLKL level at both 2 h and 6 h post-reperfusion (Fig. 6E–H). Furthermore, DFO can effectively restore the levels of LPO, MDA, and SOD, thereby reversing antioxidant status (Fig. 6I–K). These results, together with the bioinformatics analysis, indicate that iron participates in both ferroptosis and necroptosis simultaneously during IS.

### Iron effectively increases the susceptibility to necroptosis *in vitro*

**Figure 7:**
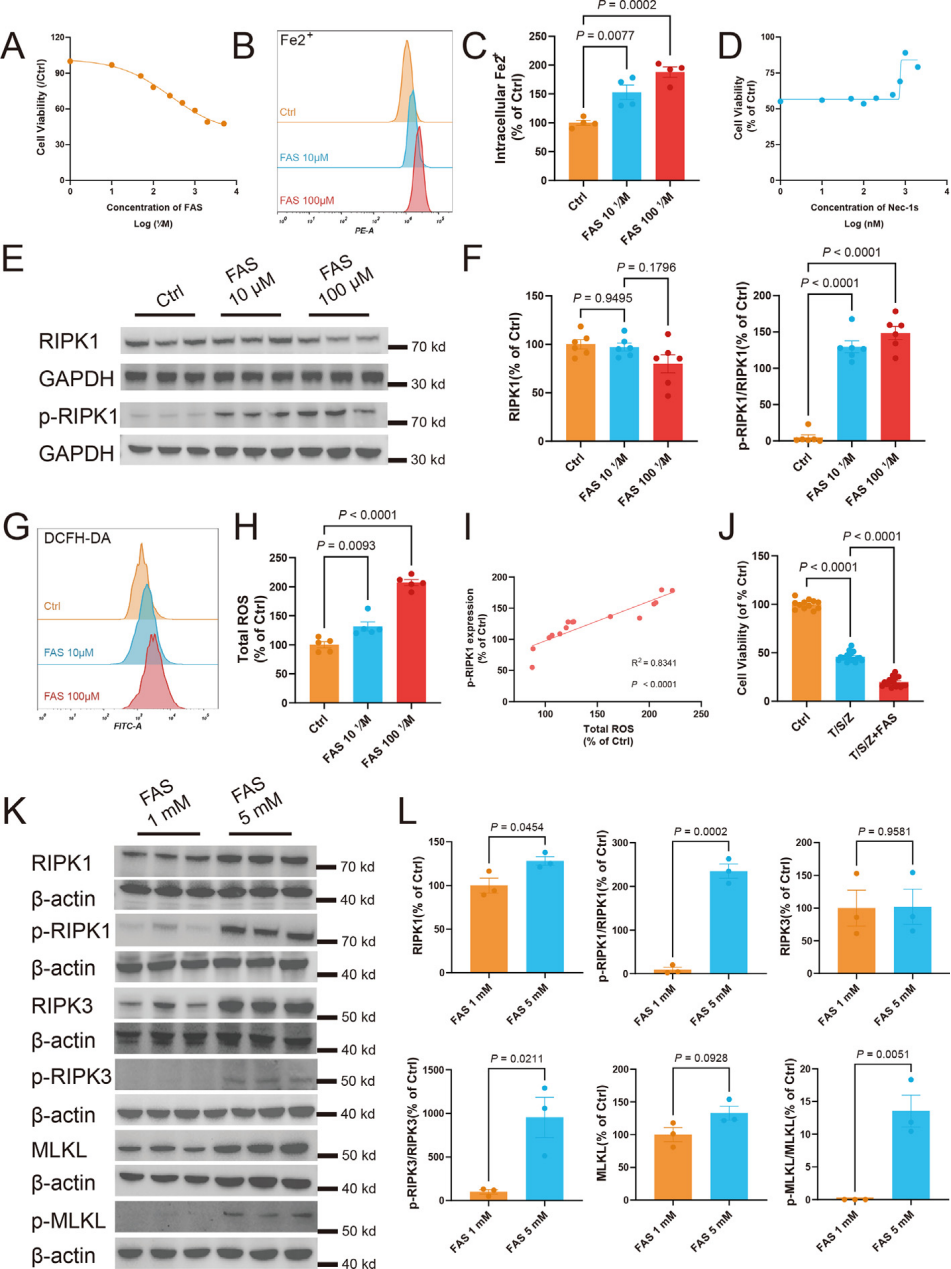
Iron effectively increases the susceptibility to necroptosis *in vitro*. **(A)** FAS cytotoxicity in N2a cells. The data presented are mean ± standard error of the mean (SEM), with *n* = 12 wells, and three independent experiments were performed. **(B)** FerroOrange staining (Intracellular Fe^2+^) in N2a cells treated with FAS (10 μM and 100 μM) for 24 h (representative histogram plot for fluorescence of Intracellular Fe^2+^). **(C)** The relative intracellular Fe^2+^ is quantified as the average fluorescence intensity observed in N2a cells following a 24-h treatment with FAS. The data presented are mean ± SEM, with *n* = 4 wells from one representative of three independent experiments. One-way ANOVA with a post-hoc Tukey test was performed. **(D)** Cell viability of N2a cells 24 h after co-treatment of FAS (500 μM) with Nec-1s. The data presented are mean ± SEM, with *n* = 6 wells, and three independent experiments were performed. **(E, F)** The levels of necroptotic biomarkers, RIPK1, and p-RIPK1, were examined in N2a cells treated with FAS (10 μM and 100 μM). Western blots were assessed using Image J and normalized to GAPDH expression. The data presented are mean ± SEM, with *n* = 6 per group. One-way ANOVA with a post-hoc Tukey test was performed. **(G)** Total ROS in N2a cells treated with FAS (10 μM and 100 μM) for 24 h (representative histogram plot for fluorescence of total ROS). **(H)** The relative total ROS is quantified as the average fluorescence intensity observed in N2a cells following a 24-h treatment with FAS. The data presented are mean ± SEM, with *n* = 5 wells from one representative of three independent experiments. One-way ANOVA with a post-hoc Tukey test was performed. **(I)** The correlation between the total ROS level and p-RIPK1 expression in N2a cells following FAS treatment. **(J)** Cell viability of L929 cells 3 h after co-treatment of FAS (1 mM) with 20 ng/mL TNF-α (T), 20 nM SM-164 (S), and 20 μM Z-VAD-fmk (Z). The data presented are mean ± SEM, with *n* = 18 wells. One-way ANOVA with a post-hoc Tukey test was performed. **(K, L)** The levels of necroptotic biomarkers were examined in L929 cells treated with FAS (1 mM and 5 mM). Western blots were assessed using Image J and normalized to β-actin expression. The data presented are mean ± SEM, with *n* = 3 per group. Student's *t*-test was performed. FAS, ferrous (II) ammonium sulfate; ROS, reactive oxygen species.

Iron is necessary for the execution of ferroptosis,^34^^,^^35^ and high levels of iron increase the susceptibility to ferroptosis, which is crucial in ischemic stroke.^36^ Since iron chelation here suppressed both ferroptosis and necroptosis during IS, we next investigated how iron affects necroptosis. We supplemented N2a cell lines with varying concentrations of ferrous (II) FAS, which ultimately resulted in reduced cell viability beginning at a dosage of 50 μΜ (Fig. 7A). At a non-lethal concentration of 10 μM and a sublethal concentration of 100 μM, FAS resulted in marked elevation of intracellular Fe^2+^ using flow cytometry (Fig. 7B, C), with the substantial accumulation of lipid ROS (Fig. S3A, B). Ferroptosis inhibitor Lip-1 effectively prevented cell death induced by 500 μM FAS (Fig. S3C).

As predicted in our animal study, a necroptosis inhibitor, namely Nec-1s, effectively alleviated cell death induced by iron overload (Fig. 7D). More importantly, we observed significant increases in the phosphorylation of RIPK1 upon supplementation with non-toxic doses of FAS (Fig. 7E, F). Conversely, discernible alterations were not evident in RIPK3 and MLKL, including their phosphorylated states (Fig. S4A, B). A comparatively lower expression level of RIPK3 was unveiled in the N2A cell line (Fig. S3A, B), supporting the notion that RIPK3 expression may vary between cell lines, linking to the response to necroptosis.^37^ Therefore, while iron activated RIPK1 in N2a cells, it may not trigger necroptosis. Iron overload in N2a cells also resulted in an increased accumulation of total ROS (Fig. 7G, H), exhibiting a positive correlation with the expression of p-RIPK1 (Fig. 7I). These imply that the redox imbalance induced by iron overload could potentially facilitate the activation of necroptosis.

In addition, a necroptosis-sensitive fibroblast cell line L929 was tested with FAS supplementation, and a high dose of FAS (5 mM) induced toxicity (Fig. S4C). Through inducing classical necroptosis via the combined administration of small-molecule inducers^38^ TNF-α (Fig. S4D), the apoptosis protein antagonist SM-164 (Fig. S4E), and Z-VAD-FMK (Fig. S4F), we observed that low doses of FAS significantly increased susceptibility to necroptosis (Fig. 7J) and facilitated the activation of the RIPK1-RIPK3-MLKL pathway during the necroptosis process, increasing their phosphorylation levels (Fig. S4G, H). Furthermore, FAS independently stimulated the RIPK1-RIPK3-MLKL pathway, resulting in increased phosphorylation levels of each component (Fig. 7K, L). Low doses of FAS (1 mM) also led to the accumulation of total ROS (Fig. S4I, J). These findings provided further evidence that susceptibility to necroptosis is heightened by iron overload-induced redox imbalance.

## Discussion

**Figure 8:**
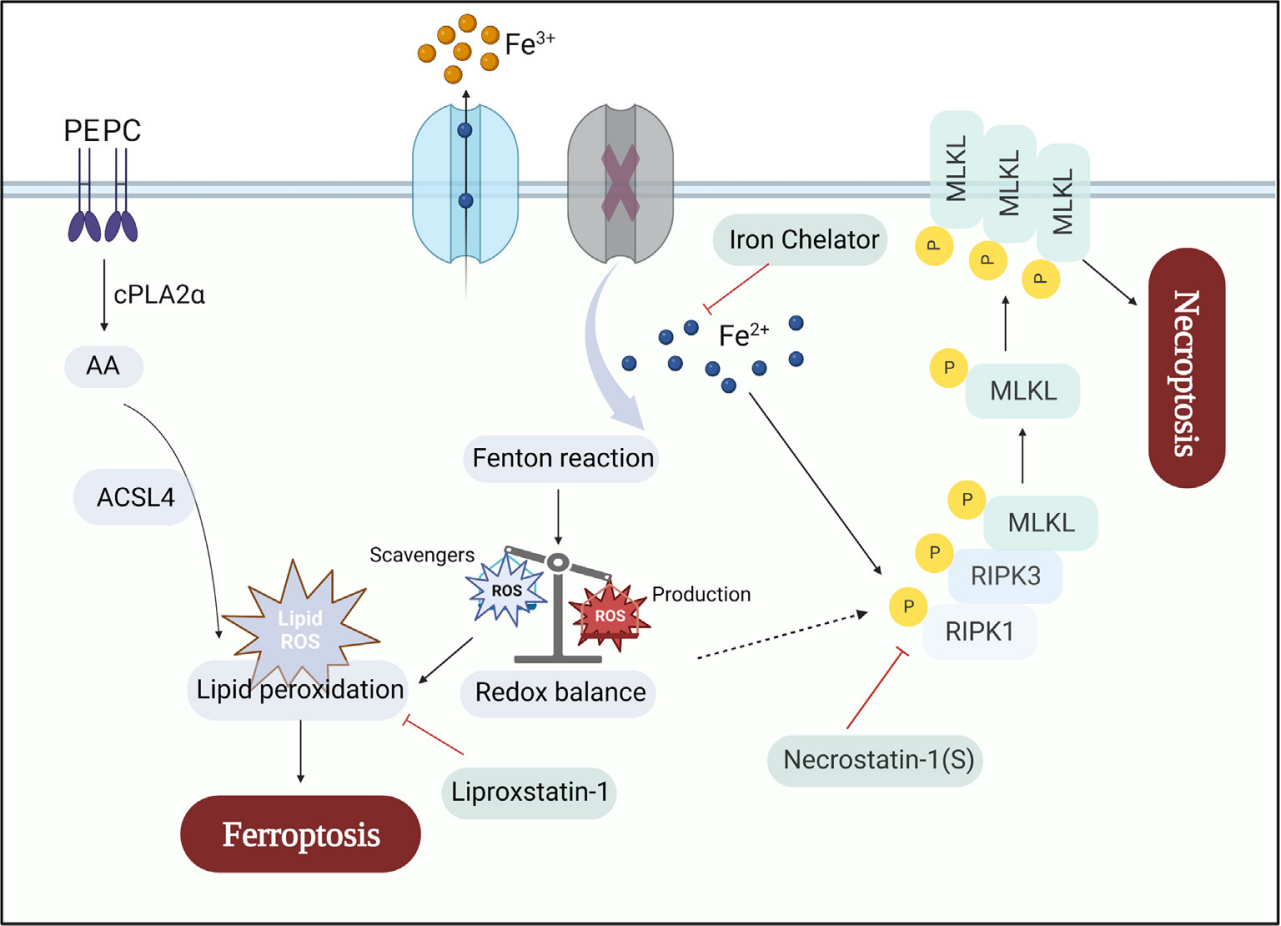
Schematic hypothesis. Ischemic stroke causes iron accumulation, disrupting the redox balance, which in turn leads to two outcomes. One of the outcomes is the increased cell susceptibility to ferroptosis, accompanied by the accumulation of lipid reactive oxygen species (ROS). Additionally, the disrupted redox balance induces the phosphorylation of RIPK1, amplifying susceptibility to necroptosis.

In recent years, several types of programmed cell death, including ferroptosis, necroptosis, and apoptosis, have been indicated in the pathogenesis of IS.^10^ Despite the development, effective treatments for IS, especially to target reperfusion-related injury, are still lacking.^6^ Most studies on IS have focused on one single cell death pathway, without considering the potential interactions between pathways. Here, we utilized unbiased bioinformatic analysis to identify differentially expressed programmed cell death-related genes and pathways post-reperfusion. Our comprehensive analysis indicates the activation of ferroptosis, necroptosis, and apoptosis post-reperfusion, as supported by immunoblot analysis. Moreover, our trend analysis of the RNA-seq data over time, combined with the findings from our pharmacological interventions, sheds light on the complex interrelationships among cell death pathways. Particularly, it highlights the previously unreported interaction of iron between ferroptosis and necroptosis (Fig. 8). These findings pave the way for further therapeutic approaches targeting reperfusion injury during IS.

Our findings here demonstrated the activation of ferroptosis, necroptosis, and apoptosis during IS. We observed that GPX4 and SLC7a11 recovered to normal levels in the late stage of reperfusion, while ACSL4 remained changed after 24 h of reperfusion. Notably, ACSL4 demonstrated substantial changes as early as 2 h post-reperfusion, highlighting its sensitivity to ischemia/reperfusion. ACSL4 was previously shown to be involved in various types of ischemia/reperfusion^39^^,^^40^ and other neurodegeneration diseases,^41^ and we previously reported^29^ that thrombin induces ACSL4-dependent ferroptosis, and the decrease of ACSL4 may be the result of protective feedback during cerebral ischemia/reperfusion. Given the absence of established biomarkers for ferroptosis, these findings imply that ACSL4 may serve as a useful biomarker for ferroptosis in the context of IS. In addition to ferroptosis, necroptosis has emerged as a significant contributor to the pathology of IS.^12^^,^^42^^,^^43^ Blocking necroptosis during IS through RIPK1 kinase inactivation, as well as deficiencies in RIPK3 and MLKL, can protect against cerebral injury.^44^^,^^45^ Pharmacological agents, including Nec-1, have been reported to inhibit necroptosis and provide neuroprotection.^46^^,^^47^ Our study found that p-RIPK1, p-RIPK3, and p-MLKL were significantly increased 2 h post-reperfusion, and p-MLKL remained elevated until 6 h of reperfusion, indicating the activation of necroptosis as an early event post-reperfusion. In contrast, we found the expression of cleaved caspase-3, a marker for apoptosis, was only detected at 6 h post-reperfusion and peaked at 24 h post-reperfusion, which is consistent with findings from other studies.^12^^,^^48^ Collectively, these findings suggest a sequential activation of ferroptosis and necroptosis preceding apoptosis during IS, emphasizing the significance of exploring the interplay between ferroptosis and necroptosis.

Under ischemic conditions, neuronal necroptosis may transit into apoptosis following the reduction of TAK1, highlighting the interplay between necroptosis and apoptosis during IS.^12^ Here we have discovered an interplay between ferroptosis and necroptosis. We found that Lip-1, a commonly used inhibitor of ferroptosis via blocking lipid peroxidation, suppressed both ferroptosis and necroptosis, mainly via effects on ACSL4 and p-MLKL expression. Analysis of protein–protein interaction networks has identified iron as a potential link between ferroptosis and necroptosis. It is known that disturbances in iron homeostasis play a key role in the pathogenesis of neuronal damage following ischemic injury.^16^^,^^36^ In the current study, DFO, an iron chelator, was shown to suppress both ferroptosis and necroptosis. Significantly, our *in vitro* experiments indicate that iron may increase susceptibility to necroptosis by augmenting the pathway of the pivotal executor RIPK1-RIPK3-MLKL within the necroptotic signaling cascade. Additionally, the necroptosis inhibitor Nec-1s effectively attenuated iron-induced cell death. Therefore, targeting the excess iron pool may be an effective strategy for ischemic reperfusion injuries.

Interestingly, Nec-1, as a necroptosis inhibitor,^47^ was also reported to block ferroptosis.^49^ One study revealed that PEBP1 acts as a switch between ferroptosis and necroptosis by inhibiting pro-necroptotic RIPK3 activity while activating 15-LOX to produce pro-ferroptotic HpETE-PE signals after irradiation and brain trauma.^50^ Moreover, the depletion of GPX4 offered initial evidence of inducing RIPK3-dependent necroptosis in mouse erythroid precursors,^51^ while HSP90 might serve as a common regulatory node for both ferroptosis and necroptosis.^38^^,^^52^ These compelling findings suggest the potential interplay between ferroptosis and necroptosis. Here, administering Nec-1 promotes the expression of ACSL4 at 2 h of reperfusion but suppresses it at 6 h. We speculate that cells might have suffered a non-fatal ferroptosis during the early stages of ischemic-reperfusion injury. Since necroptosis is mediated by active mechanisms of execution,^53^ the necroptosis inhibitor Nec-1 suppresses necroptosis but promotes non-fatal ferroptosis, which may occur to maintain a normal cell cycle and avoid internal and external stimuli. During the later stage of reperfusion, non-fatal ferroptosis can potentially transit into fatal ferroptosis, and there is a possibility that Nec-1 can rescue this period of ferroptosis, possibly due to its reported antioxidant properties.^49^^,^^54^

Iron is a pro-oxidant metal that catalyzes the formation of free radicals through the Fenton reaction, and acts as an essential cofactor in ROS-producing enzymes, contributing to regulated cell death that is dependent on ROS.^55^^,^^56^ Our findings have unequivocally shown that even at non-lethal concentrations, iron initiates the accumulation of ROS. This is important since iron accumulation has been observed in several neurological disorders including IS,^11^^,^^16^^,^^36^ as well as in aging brains.^57^^,^^58^ Our findings here indicate that such accumulation, if not toxic already, can still increase the susceptibility of cell deaths via the accumulation of ROS. Indeed, several studies have reported that ROS activated by iron can induce necroptosis in different types of cells.^55^^,^^59^ Iron also can generate lipid peroxides, thereby initiating ferroptosis.^56^^,^^60^ The accumulation of ROS provides evidence that the delicate balance between ROS induction and scavenging has been disrupted, leading to the inability of normal cellular redox balance to be maintained.^61^ Lip-1 and DFO act as inhibitors of ferroptosis and share a common mechanism for regulating redox balance. Surprisingly, in addition to its established anti-necroptotic properties, Nec-1 is reported to be an antioxidant,^49^ which is consistent with our findings here. These results highlight the importance of iron regulation and redox balance in the interplay between ferroptosis and necroptosis.

Here, we have demonstrated that iron chelation effectively reduces neurological damage by inhibiting both ferroptosis and necroptosis, focusing on post-reperfusion ischemic lesion formation and neural function assessment. While the mechanisms of iron in ferroptosis are well-acknowledged, we found here that iron activates the crucial RIPK1-RIPK3-MLKL pathway in necroptosis by impacting the redox balance. This study did not explore whether the increased susceptibility to ferroptosis and necroptosis induced by iron occurs via a shared or distinct pathway. Additionally, it did not explore the potential involvement of new proteins or molecules in this process. We also were unable to utilize non-invasive *in vivo* imaging techniques for a more precise understanding of lesion progression. Nevertheless, we introduce the novel concept of metal ions in necroptosis, providing a fresh perspective on the potential clinical efficacy of the iron chelator DFO and further highlighting its significance in clinical applications.

## Author contributions

Peng Lei and Qing-Zhang Tuo conceived and raised funds for the study. Peng Lei supervised the overall project. Qing-Zhang Tuo, Bin Du, and Junfen Wei carried out animal experiments. Zijie Deng, Kang Chen, and Ying Cheng performed cell biological experiments. Bin Du analyzed the transcriptome database. Zhangzhong Yang, Jie Meng, and Liuyao Zhou prepared pharmacological experiments. Bin Du, Xin Tian, and Peng Lei integrated the data and wrote the drafts of the manuscript. All authors edited and approved the manuscript.

## Funding

This work was supported by the National Key Research and Development Program of China (No. 2021YFC2500100), 1.3.5 Project for Disciplines of Excellence of West China Hospital of Sichuan University (No. ZYYC23016), the Key Research Projects of Sichuan Province, China (No. 24SYSX0093), Major Science & Technology Program of Sichuan Province, China (No. 2022ZDZX0021), the National Clinical Research Center for Geriatrics, West China Hospital, Sichuan University (China) (No. Z2023LC005), the Natural Science Foundation of Sichuan Province, China (No. 2022NSFSC1509), and the Fundamental Research Funds for the Central Universities (China) (No. 2023SCU12074).

## Data availability

RNA-seq (syn51873262) datasets are available at Synapse (https://www.synapse.org/#!Synapse:syn51873262/wiki/622822). To access the data, a data use agreement is needed.

## Conflict of interests

Both Peng Lei and Xin Tian serve as members of the editorial broad for Genes & Diseases but were not involved in the editorial process of this article. All other authors declare that they have no competing interests.
