# Supplementary material for: Iron promotes both ferroptosis and necroptosis in the early stage of reperfusion in ischemic stroke
Source: Genes Dis. 2024 Mar 8;11(6):101262. doi: 10.1016/j.gendis.2024.101262 (PMC11402992; doi:10.1016/j.gendis.2024.101262)
Supplement: Multimedia component 1 [file mmc1.docx]

**Supplemental materials for**

**This supplemental material includes:**

**Figure S1:**An analysis of the KEGG pathways of differentially expressed genes at various reperfusion time points in ischemic stroke

**Figure S2:** Reperfusion is the key factor responsible for the activation of ferroptosis and necroptosis in ischemic stroke

**Figure S3:** Iron effectively triggers ferroptosis *in vitro*

**Figure S4:** Iron effectively increases the susceptibility to necroptosis *in vitro*

**
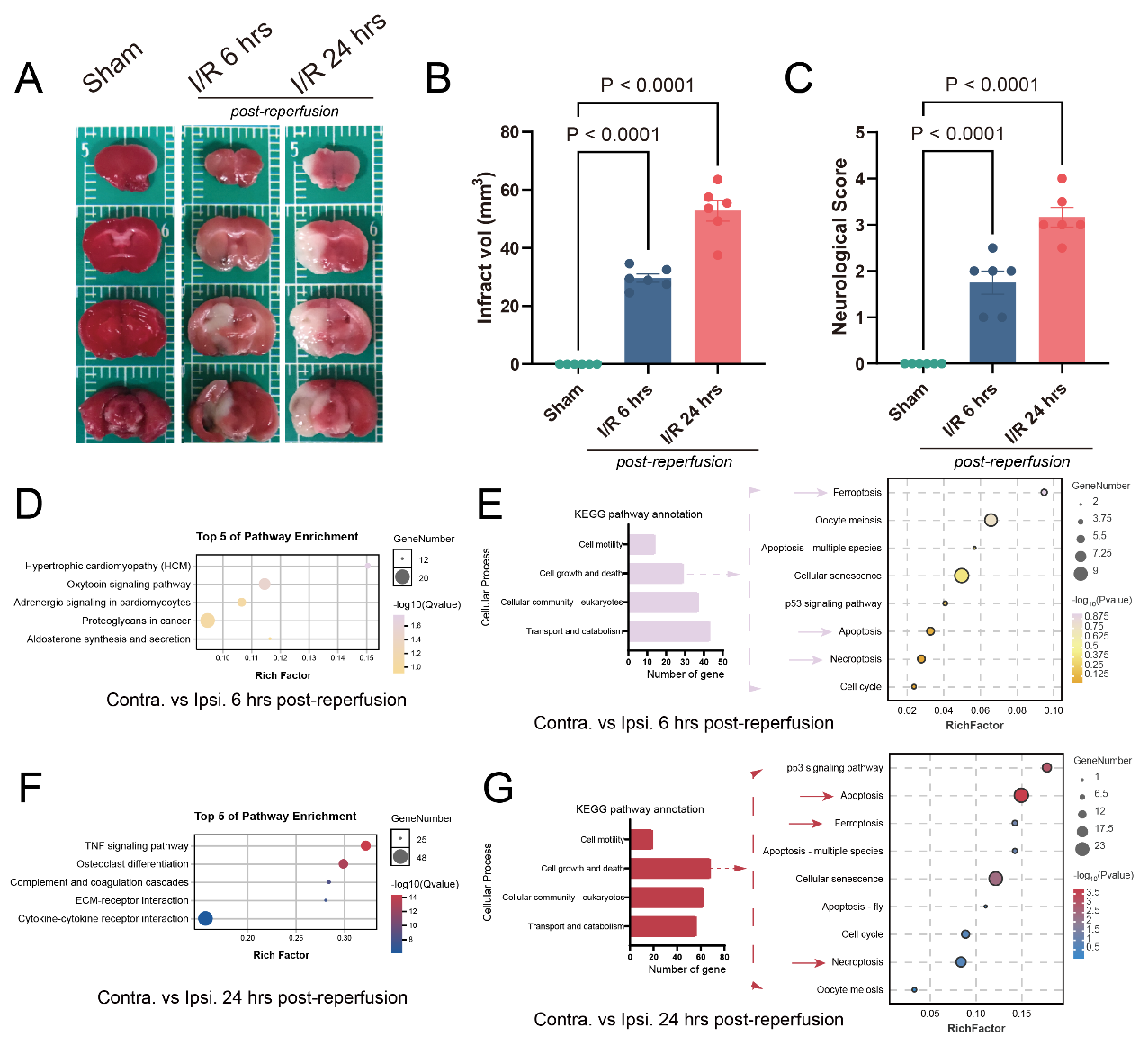
**

**Figure S1.** An analysis of the KEGG pathways of DEGs at various reperfusion time points in ischemic stroke

**(A, B)** Representative TTC-stained serial brain sections of mice at 6 hrs and 24 hrs post-reperfusion, where viable tissue stains red (A). Quantification of infarction volume indicated by TTC staining using Image J (B). Data are means ± SEM, n = 6 animals per group. One-way ANOVA with a post-hoc Tukey test was performed. **(C)** Neurological scoring, with higher numbers indicating more severe impairment, was conducted at 6 and 24 hrs post-reperfusion. Data are means ± SEM, n = 6 animals per group. One-way ANOVA with a post-hoc Tukey test was performed. **(D)** Considering all pathways, the top 5 pathways were identified through an analysis of the KEGG pathways of DEGs at 6 hrs post-reperfusion in cerebral I/R injury. **(E)** A KEGG pathway analysis specifically targeting "Cell growth and death" was conducted for the DEGs at 6 hrs post-reperfusion in cerebral I/R injury. **(F)** Considering all pathways, the top 5 pathways were identified through an analysis of the KEGG pathways of DEGs at 24 hrs post-reperfusion in cerebral I/R injury. **(G)** A KEGG pathway analysis specifically targeting "Cell growth and death" was conducted for the DEGs at 24 hrs post-reperfusion in cerebral I/R injury.

**
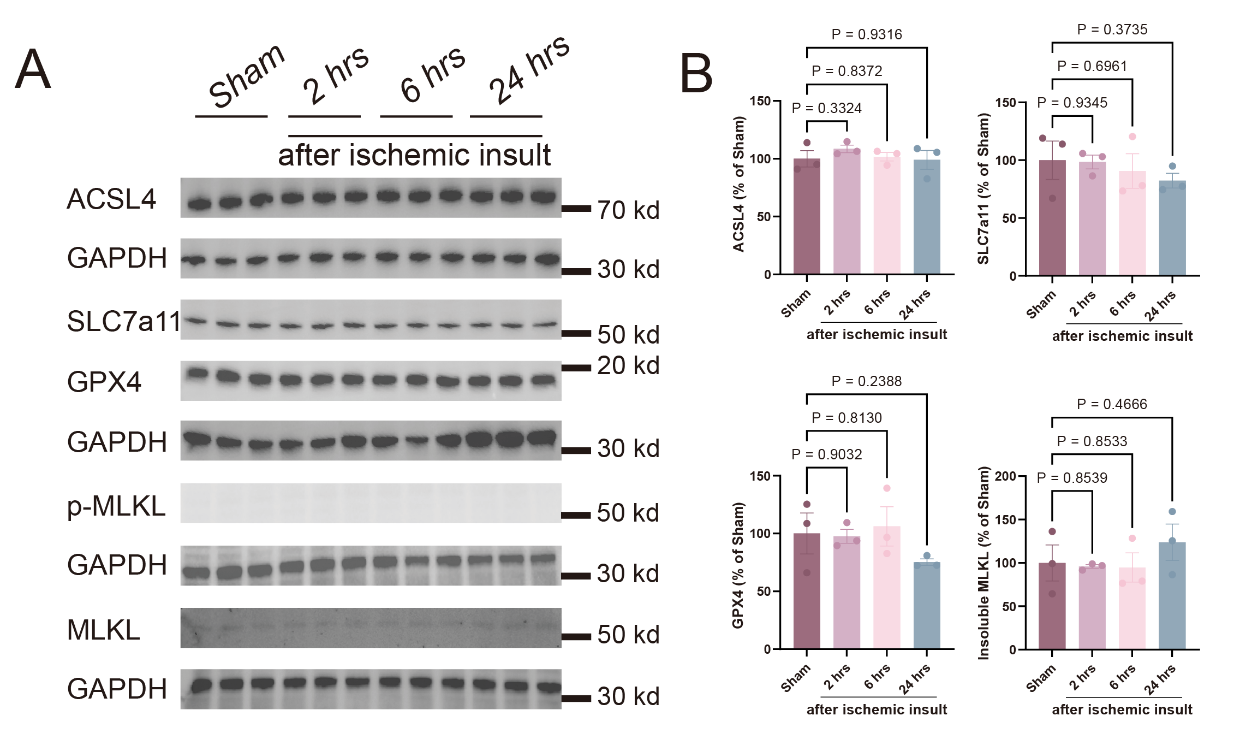
 Figure S2.** Reperfusion is the key factor responsible for the activation of ferroptosis and necroptosis in ischemic stroke

**(A, B)** The levels of ACSL4, SLC7a11, GPX4, insoluble MLKL, and insoluble p-MLKL were analyzed from the ischemic ipsilateral cortex of mice that underwent permanent occlusion at 2 hrs, 6 hrs, and 24 hrs without reperfusion (A). Western blots were quantified using Image J and normalized to GAPDH expression (B). The data presented are means ± SEM, with n = 3 animals per group. One-way ANOVA with a post-hoc Tukey test was performed.


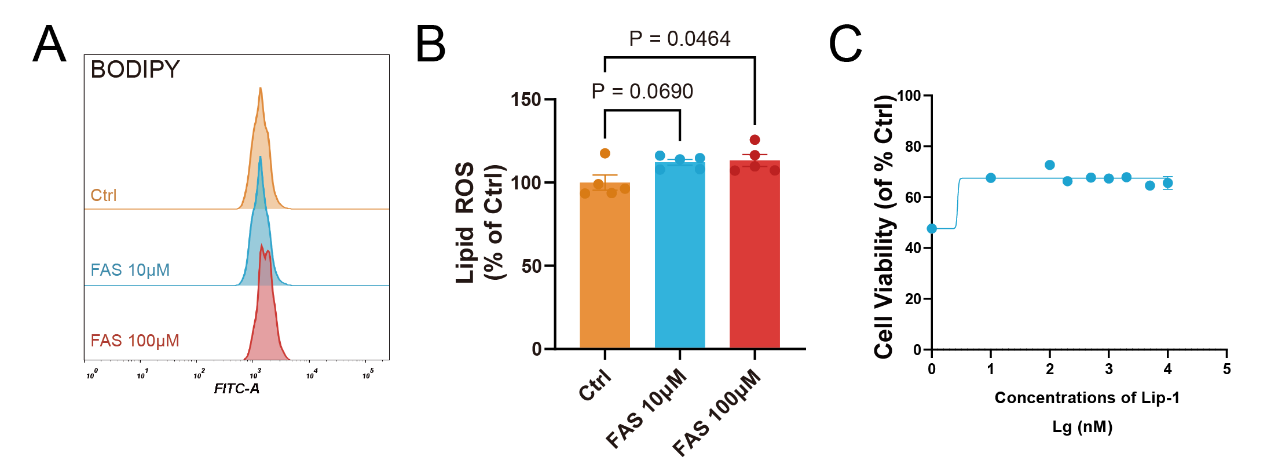


**Figure S3.** Iron effectively triggers ferroptosis *in vitro*

**(A)** Representative histogram plot showing the fluorescence of Lipid ROS in N2a cells treated with FAS (10 μM and 100 μM) for 24 hrs. **(B)** The relative Lipid ROS is quantified as the ratio of oxidized to reduced BODIPY-C11 mean fluorescence intensity in N2a cells following a 24 hrs treatment with FAS. Data are means ± SEM, n = 5 wells from one representative of three independent experiments. One-way ANOVA with a post-hoc Tukey test was performed. **(C)** Cell viability of N2a cells 24 hrs after FAS (500 μM) with Lip-1 co-treatment. Data are means ± SEM, n = 12 wells, and three independent experiments were performed.

**
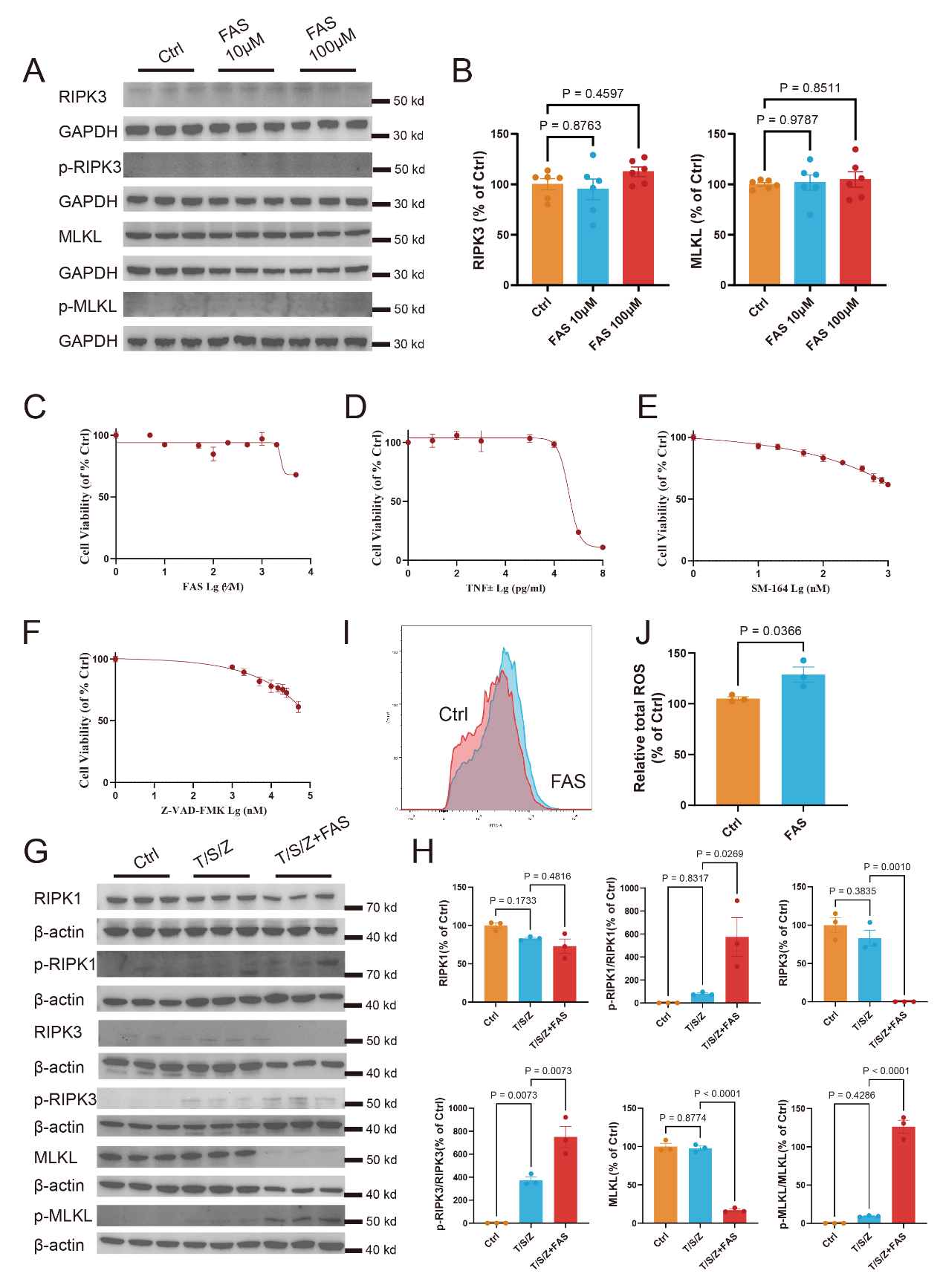
**

**Figure S4 Iron effectively increases the susceptibility to necroptosis *in vitro***

**(A, B)** The levels of necroptotic biomarkers, RIPK3, p-RIPK3, MLKL and p-MLKL were examined in N2a cells treated with FAS (10 μM and 100 μm) (A). Western blots were assessed using Image J and normalized to GAPDH expression (B). The data presented are means ± SEM, with n = 6 per group. One-way ANOVA with a post-hoc Tukey test was performed. **(C)** FAS cytotoxicity in L929 cells. Data are means ± SEM, n = 6 wells, and three independent experiments were performed. **(D-F)** TNF-α (D), SM-164 (E) and Z-VAD-fmk (F) cytotoxicity in L929 cells. Data are means ± SEM, n = 6 wells, and three independent experiments were performed. **(G, H)** The levels of necroptotic biomarkers were assessed in L929 cells treated with FAS (1 mM) and TNF-α (T), 20 nM SM-164 (S), and 20 μM Z-VAD-fmk (Z) co-treatment (G). Western blots were assessed using Image J and normalized to β-actin expression (H). The data presented are means ± SEM, with n = 3 per group. One-way ANOVA with a post-hoc Tukey test was performed. **(I)** Total ROS in L929 cells treated with FAS (1 mM) for 24 hrs (representative histogram plot for fluorescence of Total ROS). **(J)** The relative Total ROS is quantified as the average fluorescence intensity observed in L929 cells following a 24 hrs treatment with FAS. Data are means ± SEM, n = 3 wells from one representative of three independent experiments. Student's *t*-test was performed.
